# Urgent Reno-Hepatic Artery Bypass for Acute on Chronic Mesenteric Ischemia Following Robotic Low Anterior Resection for Rectal Cancer in a Patient with Severe Stenosis of the Hepatosplenomesenteric Trunk

**DOI:** 10.70352/scrj.cr.26-0325

**Published:** 2026-11-06

**Authors:** Kazumasa Hanada, Ayako Nishiyama, Natsumi Fukuhara, Haruhiko Okada, Hidehisa Yamada

**Affiliations:** 1 Department of Vascular Surgery, Saiseikai Kawaguchi General Hospital, Kawaguchi, Saitama, Japan; 2 Department of Surgery, Saiseikai Kawaguchi General Hospital, Kawaguchi, Saitama, Japan

**Keywords:** acute mesenteric ischemia, reno-hepatic artery bypass, hepatosplenomesenteric trunk

## Abstract

**INTRODUCTION:**

Acute mesenteric ischemia, including acute exacerbation of chronic mesenteric ischemia (CMI), is a life-threatening condition associated with a 50% in-hospital mortality risk and requires immediate diagnosis, revascularization, and resection of necrotic organs. In colorectal surgery, mesenteric artery stenosis can lead to anastomotic leakage which increases morbidity and mortality. Here, we describe a case of acute exacerbation of CMI caused by severe stenosis of the hepatosplenomesenteric trunk (HSMT) and ligation of the left colic and inferior mesenteric arteries in an earlier rectal surgery that was successfully treated with urgent reno-hepatic artery bypass.

**CASE PRESENTATION:**

An 80-year-old woman was referred to our department with acute exacerbation of CMI after multiple procedures for rectal cancer. Contrast-enhanced CT revealed severe stenosis with calcification at the origin of the HSMT and hypoenhancement of the right hepatic lobe, spleen, and small intestine. Percutaneous antegrade endovascular stenting failed because the lesion was severely calcified. We opted to perform urgent open surgery, including revascularization and resection of the necrotic organs. A bypass from the right renal artery to the proper hepatic artery was selected because of the high risk of failure associated with retrograde open mesenteric stenting, the limited length of available saphenous vein, the risk of anastomotic failure secondary to infection, the patient’s poor general condition, and the degree of calcification precluding aortoiliac clamping. Postoperatively, the patient developed Takotsubo cardiomyopathy and disseminated intravascular coagulation, both of which were successfully managed and resolved in the ICU. On POD 24, the patient underwent duodeno-jejunal anastomosis, ileostomy closure, and colostomy. The patient tolerated oral intake well 80 days after arterial bypass surgery and was discharged on POD 147 on single antiplatelet therapy.

**CONCLUSIONS:**

Although hepato-renal artery bypass is typically used for renal revascularization, reno-hepatic bypass can be useful for mesenteric revascularization in patients with anatomical variations, peritonitis, or severe aortoiliac calcification.

## Abbreviations

AMI
: acute mesenteric ischemia
CHA
: common hepatic artery
CMI
: chronic mesenteric ischemia
eGFR
: estimated glomerular filtration rate
EVAR
: endovascular aortic repair
EVT
: endovascular therapy
HSMT
: hepatosplenomesenteric trunk
ICG
: indocyanine green
IMA
: inferior mesenteric artery
LCA
: left colic artery
LHA
: left hepatic artery
PHA
: proper hepatic artery
RA
: renal artery
ROMS
: retrograde open mesenteric stenting
SMA
: superior mesenteric artery

## INTRODUCTION

AMI, including acute exacerbation of CMI, is a life-threatening condition that is associated with a 50% in-hospital mortality risk and requires immediate diagnosis, revascularization, and resection of necrotic organs.^1)^ CMI may be misdiagnosed because of atypical complaints such as constant abdominal discomfort, nausea, vomiting, diarrhea, or constipation.^1)^ In colorectal surgery, CMI or mesenteric artery stenosis can lead to anastomotic leakage, which increases morbidity and mortality.^2)^ Here, we describe a case of acute exacerbation of CMI caused by severe stenosis of the HSMT and ligation of the LCA and IMA in an earlier rectal surgery, successfully treated with an urgent reno-hepatic artery bypass and resection of the necrotic organs. The patient consented to publication of the details and images pertaining to this case.

## CASE PRESENTATION

An 80-year-old woman with hematochezia and postprandial epigastric pain was admitted to the department of gastroenterology for further investigation and treatment. She was taking oral medications for hypertension. She was 155 cm tall and weighed 50 kg (BMI, 20.8). No weight loss occurred within the past year. Her abdomen was soft and nondistended. Routine blood test results were unremarkable. The eGFR was 56.7 mL/min/1.73 m^2^. Her medical history was negative for rheumatological or systemic diseases. There were no clinical signs of inflammation or infection that were observed. Colonoscopy and contrast-enhanced CT revealed rectal cancer. Electrocardiography did not indicate atrial fibrillation. Robot-assisted low anterior resection was performed at the department of gastrointestinal surgery. During the operation, the LCA and IMA were ligated. In the ICG fluorescence assessment after division of the IMA and LCA, blood flow was not observed in the sigmoid colon but was confirmed in the descending colon; therefore, the splenic flexure was mobilized to perform the anastomosis. Following initiation of a postoperative diet, the patient developed abdominal pain, diarrhea, and emesis. Anastomotic leakage was detected by radiographic contrast enema on POD 8. Laparoscopic ileostomy was performed on POD 15. An ileostomy revealed a dusky discoloration, which worsened over time. On POD 22, blood tests showed an increased white blood cell count (19.9 × 10^9^/L) and increased level of C-reactive protein (15.18 mg/dL), lactate (5.54 mmol/L), and procalcitonin (3.92 ng/mL). Contrast-enhanced CT revealed severe stenosis with calcification at the origin of the HSMT and hypoenhancement of the right hepatic lobe, spleen, and small intestine (**Fig. 1**). The bifurcation of the hepatosplenic trunk and SMA was patent. The left gastric artery branched independently from the abdominal aorta and gave rise to an aberrant LHA. This anatomical variation preserved perfusion in the stomach and left lobe of the liver. The patient was referred to our department with a diagnosis of acute exacerbation of CMI. Her blood pressure, pulse rate, and percutaneous oxygen saturation were 189/88 mmHg, 98 beats/min, and 98%, respectively. The abdomen was mildly distended and soft, with tenderness primarily localized in the epigastrium. There were no signs of peritoneal irritation.

**Fig. 1:**
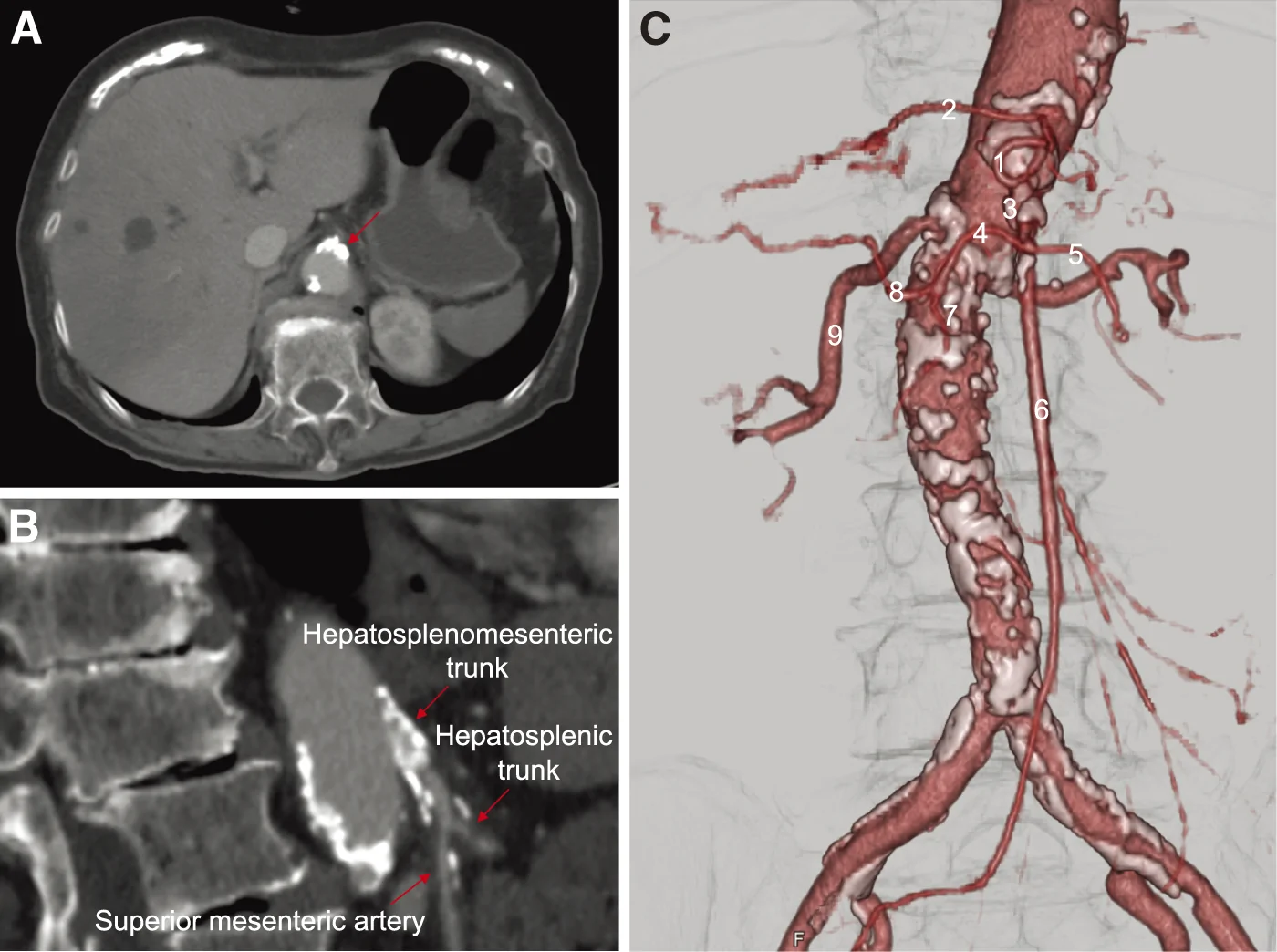
CT showing a calcified lesion at the origin of the HSMT (indicated by the arrow) with (**A**) axial and (**B**) sagittal images. (**A**) CT showing hypoenhancement of the right lobes of the liver and spleen. (**C**) 3D reconstruction of 1) left gastric artery; 2) aberrant LHA; 3) HSMT, 4) CHA, 5) splenic artery; 6) SMA; 7) gastroduodenal artery; 8) PHA; and 9) right RA. CHA, common hepatic artery; HSMT, hepatosplenomesenteric trunk; LHA, left hepatic artery; PHA, proper hepatic artery; RA renal artery; SMA, superior mesenteric artery

We initially decided to perform an emergency endovascular stenting of the HSMT under local anesthesia in the angiography suite, rather than proceeding with an open or hybrid operation, because a hybrid operating room was unavailable. Percutaneous antegrade endovascular stenting failed as the 15-mm severely calcified HSMT lesion could not be crossed despite multiple access sites, including bilateral femoral and left brachial arteries. Angiography revealed that the HSMT was not completely occluded and that distal blood flow was preserved (**Fig. 2**). Although heparinization and vasodilators were initiated in the ICU, the ileostomy became increasingly dusky (**Fig. 3A**). Twenty hours after the referral, blood tests showed increased levels of aspartate aminotransferase (327 IU/L), alanine aminotransferase (238 IU/L) and increased level of white blood cell count, C-reactive protein, lactate, and procalcitonin. CT revealed portal venous gas, which was not present in the previous scan.

**Fig. 2:**
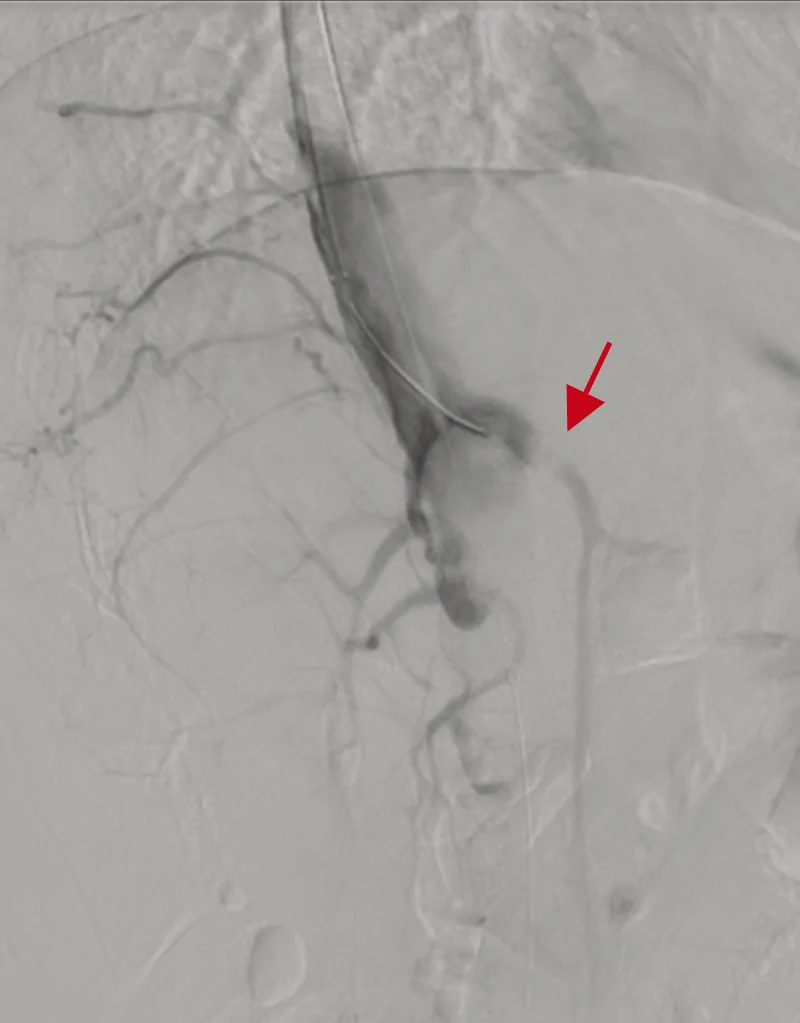
Intraoperative angiography shows a calcified stenotic lesion at the origin of the HSMT (arrow). HSMT, hepatosplenomesenteric trunk

**Fig. 3:**
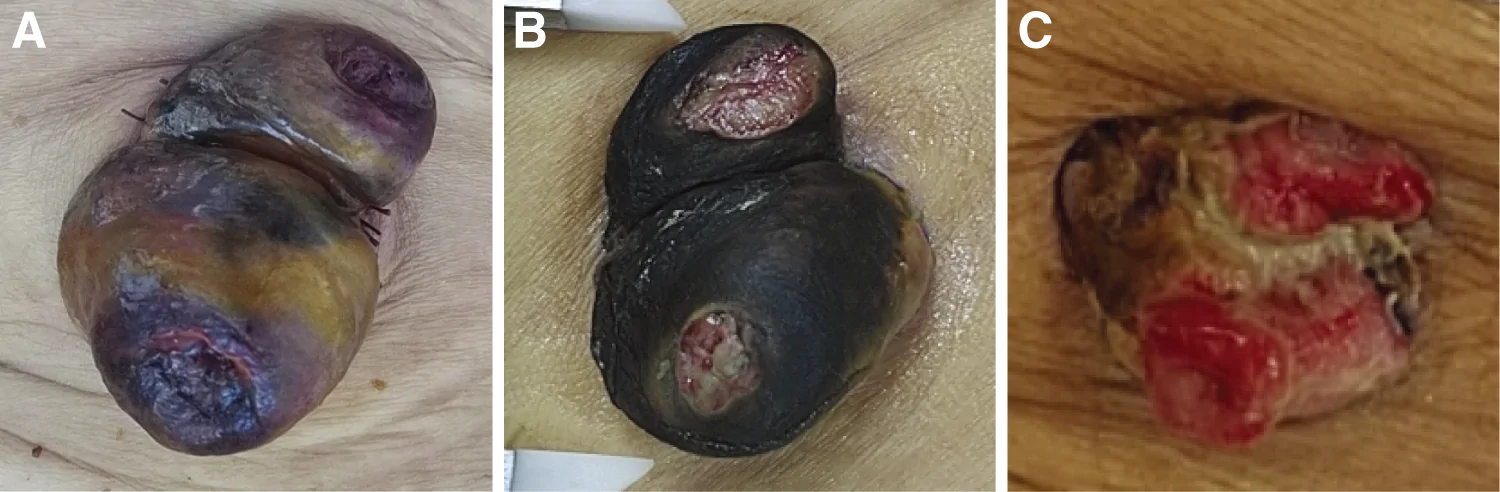
Macroscopic findings of the ileostomy (**A**) after failed endovascular stenting, (**B**) 7 days after renohepatic artery bypass, and (**C**) 21 days after bypass and following debridement.

We opted to perform an urgent open surgery, including revascularization and resection of the necrotic organs. We decided on the bypass from the right RA to the PHA for 4 reasons. Through this approach, blood flow to the SMA can be secured via the following pathway: the right RA, the bypass graft, the PHA, the CHA, the distal portion of the HSMT, and finally the SMA. First, ROMS was considered difficult due to lack of adequate equipment at our facility and high risk of failure owing to severe calcification. Second, the available saphenous vein was not long enough from the iliac artery to the hepatic artery. Third, proximal anastomosis to the iliac artery carried a risk of anastomotic failure secondary to infection. Fourth, the patient’s poor general condition and calcification did not allow aortoiliac clamping.

Under general anesthesia, a midline laparotomy was performed, which revealed turbid ascites and necrosis of the gallbladder, 200 cm of the small intestine from the ligament of Treitz, and the transverse colon. The right RA was exposed following the Kocher maneuver. The hepatoduodenal ligament was dissected, and proper hepatic, gastroduodenal, and right gastric arteries were identified and exposed. A 12-cm segment of the great saphenous vein, with a diameter of at least 2.5 mm was harvested from the right thigh. Other vein segments were unsuitable for grafting due to varicose changes. The bypass was constructed as an end to side anastomosis of the reversed vein graft between the right RA and PHA (**Fig. 4A**). Flow meter measurements revealed a flow rate of 280 mL/min in the proximal right RA, 215 mL/min in the graft, 180 mL/min in the PHA, and a retrograde flow of 211 mL/min in the CHA. After the bypass, the demarcation of the necrotic bowel was clear. ICG fluorescence assessment was not performed due to institutional constraints. The necrotic organs were resected by gastrointestinal surgeons without reconstruction, and the graft was placed on the gallbladder bed without tension (**Fig. 4B**). Intraoperative blood loss was 468 mL, and the operative time was 314 min.

**Fig. 4:**
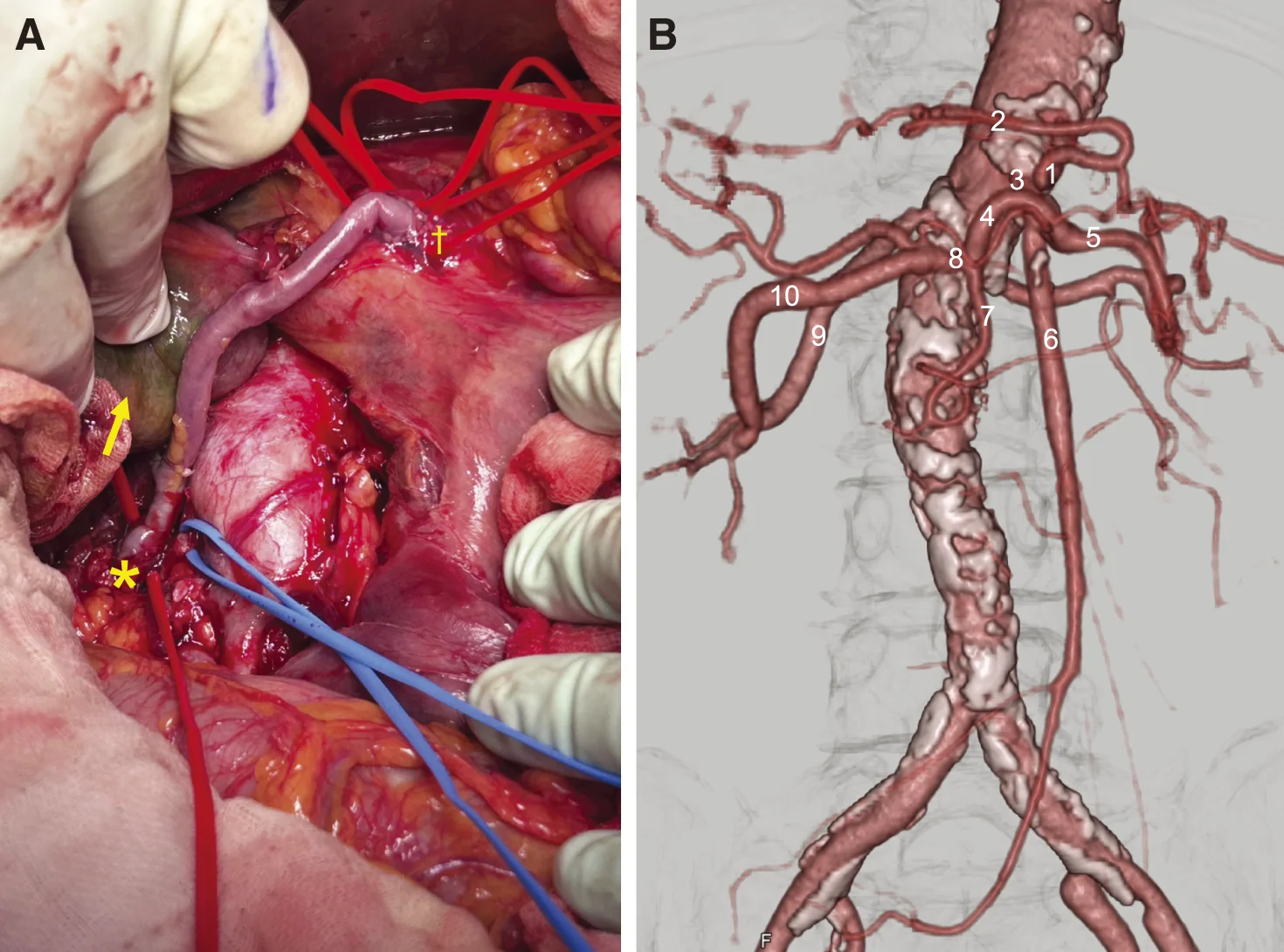
(**A**) Intraoperative finding shows the reno-hepatic bypass between the right RA (*) and PHA (†) before the resection of necrotic gallbladder (indicated by the arrow). A reversed saphenous vein graft was used. (**B**) 3D reconstruction of 1) left gastric artery, 2) aberrant LHA; 3) HSMT, 4) CHA, 5) splenic artery; 6) SMA; 7) gastroduodenal artery; 8) PHA; 9) right RA; and 10) reversed saphenous vein graft. CHA, common hepatic artery; HSMT, hepatosplenomesenteric trunk; LHA, left hepatic artery; PHA, proper hepatic artery; RA, renal artery; SMA, superior mesenteric artery

During the postoperative course, the patient developed Takotsubo cardiomyopathy (peak brain natriuretic peptide level of 1588.3 pg/mL) and disseminated intravascular coagulation; however, these conditions were successfully managed and resolved in the ICU. The discoloration of the ileostomy gradually improved (**Fig. 3B** and **3C**). On POD 24, gastrointestinal surgeons performed a duodeno-jejunal anastomosis, closed the ileostomy, and created colostomy. The length of the residual small intestine was >100 cm. The patient tolerated oral intake well 80 days after arterial bypass surgery and was discharged on POD 147 on single antiplatelet therapy.

Although contrast-enhanced CT at 2 weeks revealed hypoperfusion of the right kidney, suggestive of renal steal, follow-up imaging at 2 months showed resolution (**Fig. 5**). The eGFR was consistently preserved. CT confirmed good patency of the reno-hepatic artery bypass graft, with well-preserved blood flow to the distal side of the HSMT (**Fig. 4B**).

**Fig. 5:**
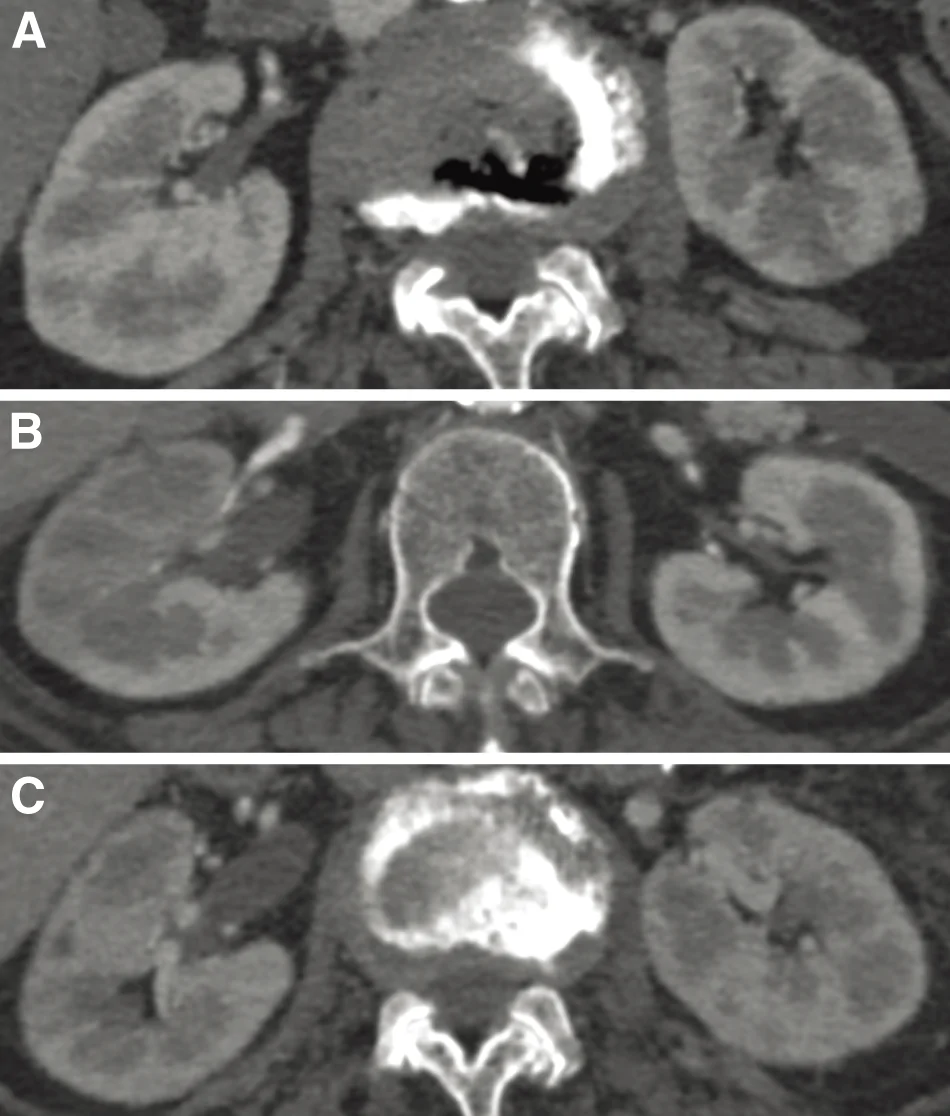
CT showing changes in kidney enhancement. (**A**) Preoperative enhancement is similar in both kidneys. (**B**) Two weeks after surgery, the enhancement of the right kidney is low. (**C**) At 2 months, the enhancement in both kidneys is again equal.

## DISCUSSION

In AMI and exacerbated or symptomatic CMI, SMA revascularization is the priority.^1)^ In cases of AMI or acute exacerbation of CMI caused by atherosclerosis or thrombosis, as in the present case, the treatment strategy depends on the presence of intestinal necrosis. If there are no clear signs of intestinal necrosis, EVT with a balloon-expandable covered or bare-metal stent should be performed, followed by close observation. If necessary, subsequent resection of the necrotic bowel should be performed. If signs of intestinal necrosis are present, revascularization and resection of the necrotic bowel should be performed in a hybrid operating room. Revascularization options include antegrade percutaneous stenting, ROMS, or surgical bypass.^1)^ In a retrospective study of AMI, 6-month primary patency in AMI was the highest with open surgery (not limited to surgical bypass) at 100%, compared with 87.3% for EVT and 80% for ROMS.^3)^

Open revascularization includes bypass distal to the occlusion or stenosis, thromboendarterectomy, and ROMS.^1)^ We did not perform thromboendarterectomy because of its high surgical invasiveness. ROMS was contraindicated because of facility constraints and the high risk of procedural failure or restenosis associated with calcification. Bypass planning, in general, involves selecting the approach (transperitoneal vs. retroperitoneal), conduit (vein vs. prosthetic), graft configuration (antegrade vs. retrograde), and source of inflow (aortic vs. iliac).^1)^ In our case, several surgical constraints dictated the operative plan. A transperitoneal approach was necessary for the resection of necrotic organs, and a venous conduit was selected because of the high risk of infection. Although a retrograde graft configuration was preferred considering the surgical invasiveness and extensive aortoiliac calcification, determining the source of inflow was challenging. Furthermore, the great saphenous vein was not long enough to reach the iliac artery as an inflow source. Consequently, we opted to use the right RA as the source of inflow because renal function was normal.

HSMT is a rare anatomical variant in which the CHA, splenic artery, and SMA share a common origin. Reported prevalence ranges from 0% to 1.2%, with a systematic review indicating a global prevalence of 0.45%. Its association with an aberrant LHA (Adachi Type III group 19 classification) is extremely rare.^4–6)^ It is essential to recognize such anatomical anomalies in vascular surgery, EVT, and digestive surgery in order to ensure appropriate preoperative planning.^4,6,7)^ In our case, exacerbation of ischemia in the territory of the HSMT was caused by ligation of the LCA and IMA, which had previously maintained collateral blood flow to the area. Retrospectively, the preoperative CT for rectal cancer showed that the origin of the HSMT was calcified and stenotic and that the arc of Riolan was dilated to approximately 2.3 mm, a finding consistent with SMA stenosis.^8)^ Reno-hepatic artery bypass proved to be an effective revascularization strategy for near occlusion confined to the origin of the HSMT.

Reno-hepatic artery bypass (or hepato-RA bypass) is an established option for renal revascularization.^9)^ In the current endovascular era, hepato-RA bypass remains a viable option for patients who are not candidates for endovascular renal revascularization, as well as for cases involving debranched EVAR or RA occlusion following EVAR.^9,10)^ In hybrid procedures for hepatic or pancreaticoduodenal artery aneurysms (comprising percutaneous coil embolization and open surgical revascularization), reno-hepatic artery bypass is a simple and effective technique.^11,12)^ A key advantage of this procedure is the short graft length, which minimizes the risk of graft compression, kinking, or twisting. One potential complication of bypass is renal steal, which can lead to renovascular hypertension or impaired renal function, particularly in the presence of increased recipient-side blood flow or the progression of stenosis at the origin of the RA.^13,14)^ Therefore, indications for bypass should be carefully considered. In this case, intestinal reconstruction was performed on POD 24 when the patient’s general condition stabilized and the color of the ileostomy indicated that reconstruction was feasible. This timing occurred between day 14 when renal steal was suspected and 2 months postoperatively, when the suspected findings had disappeared. Physiologically, this hemodynamic adaptation requires approximately 4 to 8 weeks, during which a donor artery completes flow-mediated outward remodeling to fully stabilize an expanded vascular territory.^15,16)^ Although waiting for further hemodynamic maturation would be ideal from a hemodynamic standpoint, we believed it was necessary to perform early intestinal reconstruction, even if it caused renal steal or right kidney ischemia, considering the patient’s nutritional status and prognosis. Consequently, the intestinal reconstruction was successful, and there were no findings suggesting renal steal during the follow-up. The patient should be closely monitored with the intention of performing percutaneous transluminal renal angioplasty if renal steal occurs or if RA stenosis is exacerbated (resulting in decreased blood flow in the territory of the HSMT).

In colorectal surgery, it is important to evaluate for stenosis or occlusion of the SMA and IMA on preoperative CT. A retrospective study revealed that stenosis exceeding 50% in the SMA or IMA on CT was associated with 6- and 2-fold higher odds of anastomotic leakage, respectively.^2)^ To the best of our knowledge, there are no prior reports or studies specifically addressing AMI following colorectal surgery. However, it is plausible that acute exacerbation of CMI can be induced by vascular ligation during colorectal surgery, similar to the risk of anastomotic leakage.

## CONCLUSIONS

A reno-hepatic artery bypass can be a useful option for mesenteric revascularization in patients with anatomical variations, peritonitis, aortoiliac calcification, or poor general condition. In a colorectal surgery, evaluation of the mesenteric arteries and their collateral pathways is important.

## DECLARATIONS

### Availability of data and materials

Not applicable as no datasets were generated or analyzed during the current study.
